# Translation, cross-cultural adaptation, and validation of the Chinese version of the 4 domain sports prom

**DOI:** 10.1186/s13018-025-05882-1

**Published:** 2025-05-19

**Authors:** Yang Sun, Shiyi Chen, Philippe Neyret, Mark Hutchinson, Filippo Migliorini, Nicola Maffulli, Carlos Górios, Sérgio Rocha Piedade

**Affiliations:** 1 Sports Medicine Fudan Un, Shanghai, China; 2 Burjeel Hospital, Abu Dhabi, UAE; 3 Department of Orthopaedic Surgery, University of Illinois at Chicago, IL Chicago, United States; 4 Department of Trauma and Reconstructive Surgery, University Hospital of Halle, Martin Luther University Halle-Wittenberg, Ernst-Grube-Street 40, Halle (Saale), 06097 Germany; 5 Department of Orthopaedic and Trauma Surgery, Academic Hospital of Bolzano (SABES-ASDAA), Via Lorenz Böhler 5, 39100 Bolzano, Italy; 6 Department of Life Sciences, Health, and Health Professions, Link Campus University, Via del Casale Di San Pio V, 00165 Rome, Italy; 7 Faculty of Medicine and Psychology, Sapienza University, Rome, Italy; 8 Centre for Sports and Exercise Medicine, The London School of Medicine and Dentistry, Mile End Hospital, Queen Mary University of London, Barts, London, E1 4DG UK; 9 School of Pharmacy and Bioengineering, Keele University School of Medicine, Thornburrow Drive, Stoke On Trent, England; 10 Centro Universitário São Camilo, Ipiranga, São Paulo, Brazil; 11 Exercise and Sports Medicine, Department of Orthopaedic, Rheumatology, and Traumatology, School of Medical Sciences, University of Campinas, UNICAMP, Campinas, Brazil

**Keywords:** Sports Medicine, Patient-reported outcome Measures, Patient outcome Assessment, Outcomes and Process Assessment

## Abstract

**Background:**

This work evaluated and validated the translation and cross-cultural adaptation of 4-Domain Sport PROM (4-DSP) into Chinese, assessing its understandability and reproducibility in all questionnaire domains for Chinese-speaking patients.

**Methods:**

Cross-sectional study, level of evidence II. Twenty patients with sports injuries who underwent surgical treatment and postoperative rehabilitation in the Sports Medicine Surgery Department of Huashan Hospital were selected to evaluate whether the translation was understandable. Then, the 4-DSP was applied to 120 patients who had undergone trauma surgical procedures. The translation and cross-cultural adaptation of 4-DSP involved 6 steps: (1) Translation, (2) Synthesis of translation, (3) Back Translation, (4) Testing of the Prefinal Version—Expert committee review, (5) Prefinal testing among the patients, and Reliability and Consistency Testing. The questionnaire was self-administered by 120 patients (53 males and 67 females; mean age: 30.41 ± 6.8 years.) who had undergone arthroscopic surgery or conservative therapy from a sports physical therapist and had 3-month to 1-year follow-up. All patients filled in the 4-DSP questionnaire without direct supervision of their trainer/coach or researcher. All data were collected and processed anonymously.

**Results:**

97% of the experts (*n* = 10) considered the translation accuracy understandable; each item and overall content validity showed 96% agreement, and the bilingual translation accuracy was rated as 98.5%, presenting a global Cronbach's alpha of 0.72.

**Conclusion:**

The Chinese cross-cultural adaptation and validation of the original English version of the 4-DSP questionnaire proved reproducible and properly understandable in all four domains. It can safely and reliably assess treatment outcomes for sports injuries in Chinese-speaking patients and is a helpful tool to collect and assess athletic population data.

**Supplementary Information:**

The online version contains supplementary material available at 10.1186/s13018-025-05882-1.

## Background

Orthopaedic surgeons and sports physicians have come to understand that the optimal way to analyze the result of treatment outcomes is by collecting data from the patient´s point of view. [1–3] Therefore, the Patient-Reported Outcomes measures, or PROMs, are valuable tools to assess and better understand the risks and benefits of a given treatment. [4–6]

PROMs are widely used to evaluate the outcome of patients undergoing trauma and orthopaedic surgery procedures, but most of them are mainly limited to specific anatomical sites such as the knee, shoulder, hip, foot, etc. [7–10] Moreover, most were developed to assess the general population rather than athletes and regular sports practitioners, resulting in a lack of well-developed PROMs for this patient´s group. [11]

Some authors have called attention to this and discussed the importance of a targeted patient-reported instrument, designed to assess the outcomes in athletes and sports practitioners [12–14]. More focused or condition-specific PROMs are designed to assess specific symptoms and functions related to a specific condition or body parts.

In 2021, the 4-Domain Sports Prom (4-DSP) was published. The 4-DSP is a PROM tailored to assess the treatment outcomes in athletic populations, allowing a universal approach regardless of the anatomical site of injury. [15] It is a 4-domain structured questionnaire, and collects athletes'data before the injury, with injury, athletes'expectations, treatment, and operative outcomes. Moreover, the 4-DSP allows to record and classify athletes'sports levels, main physical demands in sports, and clinical complaints.

Originally published in English [15], the 4-DSP has been cross-culturally validated in Portuguese, Spanish, and Italian. [16–18] The easy applicability of 4-DSP and the fact that it was well-designed to assess the treatment in the athletic population triggered the interest in expanding the 4-DSP use to other languages.

Chinese is one of the most widely spoken languages worldwide, and given the enormous number of sports practitioners and athletes in China and its relevance to sports medicine and orthopaedics, this study aims to translate and validate cross-culturally the questionnaire 4-Domain Sports PROM Chinese version.

To translate, cross-culturally adapt, and validate the questionnaire 4-Domain Sports PROM Chinese version so that it can be used reliably in Chinese, proceeding with content validation obtained through the evaluation of the instrument by a reliable and reproducible method. The specific aims of validating its use in the Chinese language are its application to the development of scientific research and health protocols to optimize the therapeutic approach, reduce costs, and evaluate the efficiency of services.

## Materials and methods

The 4-Domain Sports Prom is a PROM tailored to assess the treatment outcomes in athletic populations, allowing a universal approach regardless of the anatomical site of injury. It is a 4-domain structured questionnaire, designed to record and classify athletes'sports level, main physical demands in sports, and clinical complaints, collecting athletes'data before the injury, with injury, athletes'expectations, treatment, and operative outcomes.

### Study population

20 patients with sports injuries who underwent surgical treatment and postoperative rehabilitation in the Sports Medicine Department of Huashan Hospital were selected to evaluate whether the translation was understandable.

The research team, then recruited 120 patients who had undergone sports medicine surgery at the Department of Sports Medicine at Huashan Hospital affiliated to Fudan University. The questionnaire was self-administered by 120 patients (53 males and 67 females) who had undergone arthroscopic surgery or conservative therapy from a sports physical therapist and had 3-month to 1-year follow-up. The mean age and standard deviation (SD) was 30.41 ± 6.8 years. (Table 1).

**Table 1:** Patient Information (FAI: femoroacetabular impingement; ACL: anterior cruciate ligament; PCL: posterior cruciate ligament; MCL: medial collateral ligament)

Endpoint	Male	Female	Total
Number of Patients	53	67	120
Average age	30.3 ± 6.6	30.5 ± 7.0	30.4 ± 6.8
Conservative treatment	5	9	14
patellofemoral pain	2	0	2
ankle sprain	2	6	8
meniscus injury	1	2	3
partial ACL Tear + meniscus injury	0	1	1
Surgical treatment	48	58	106
Ankle cartilage microfracture	1	0	1
ACL artificial ligament reconstruction & meniscus repair	7	10	17
ACL artificial ligament reconstruction	6	7	13
ACL autograft reconstruction + meniscus suture	6	6	12
ACL autograft reconstruction	0	4	4
PCL reconstruction & MCL repair	1	0	1
Ankle lateral ligament repair	9	10	19
Shoulder instability labral fixation	1	1	2
Meniscus repair	7	6	13
MCL reconstruction	1	1	2
Chondroplasty of the knee	1	0	1
PCL autograft reconstruction	2	2	4
PCL artificial ligament reconstruction	1	1	2
Achilles tendon repair	1	1	2
Meniscectomy	1	3	4
Medial patellofemoral ligament reconstruction	1	1	2
Rotator cuff tear repair	1	0	1
Acromioclavicular joint fixation	1	0	1
FAI Hip Labral Repair	0	2	2
Knee synovectomy	0	1	1
Ankle synovectomy	0	1	1
ACL & PCL recontruction (artificial ligament)	0	1	1

### Methodology

The translation and cross-cultural adaptation of 4 Domain Sport PROM involves 6 steps: (1) Translation, (2) Synthesis of translation, (3) Back Translation, (4) Testing of the Prefinal Version—Expert committee review, (5) Prefinal testing among the patients, and Reliability and Consistency Testing. All patients filled in the 4-DSP questionnaire without direct supervision of their trainer/coach or researcher. All data were collected and processed anonymously.

### A. Translation and cross-cultural adaptation of 4 domain sport PROM.

The process of translation and cross-cultural adaptation of 4 Domain Sport PROM (Appendix 1) for Chinese followed the criteria of Beaton et al.[4], which is performed in the following five steps.

#### Step 1 Translation

The original questionnaire was translated into Chinese by two independent translators (a sports medicine surgeon and a sports rehabilitation specialist) who are native Chinese speakers proficient in English reading and writing.

#### Step 2 Synthesis of translation

Two independent translators discussed and integrated their own translations to obtain the first draft of the Chinese version of the questionnaire.

#### Step 3 Back translation

Two independent back translators (without medical background), both with English as their mother language and full fluency in Chinese, translated the Chinese version of 4 domain sport PROM back into English. They were not allowed access to the original version of the questionnaire. After comparing with the original version, the main researchers revised the first draft of the 4-DSP Chinese version after analyzing and discussing the obtained second Chinese version (Prefinal Version).

#### Step 4 Testing of the prefinal version—expert committee review

Ten experts in Trauma and Orthopaedic Surgery or in Sports Medicine who were not involved in the study were invited to evaluate the second draft of the Chinese version in two aspects, and the main researchers revised it according to the results of the expert correspondence. Among the 10 specialists were 5 sports medicine surgery specialists, 4 sports rehabilitation specialists, and 1 sports medicine specialist nurse. The experts from different specialists reported above participated in the questionnaire evaluation for higher expert validity. Experts were asked to answer two aspects of the questionnaire: (1) whether the translation was understandable. Experts used"yes"and"no"responses. 80% of the experts for each entry had to choose"yes". Otherwise, the translation had to be adjusted according to expert advice (Table 2). (2) Content validity evaluation of the questionnaire. The 10 experts were asked to rate how relevant the questions in the four topics in the second Chinese version of the questionnaire were to the connotation of the topics. The 4-level Likert scale was used for evaluation in this study (1 = not relevant; 2 = unable to assess relevance; 3 = relevant but needs minor alteration; 4 = very relevant and succinct). It was required that 80% of experts should select the 3/4 level for each item to make it clear that the content validity of the Chinese version of the questionnaire is reasonable (Table 3).

**Table 2:** Expert evaluation of translation accuracy

4 Domain Sport PROM items	Number of “Yes”	Number of “No”	“Yes” Ratio
Item 1	10	0	1.0
Item 2	9	1	0.9
Item 3	10	0	1.0
Item 4	10	0	1.0
Item 5	10	0	1.0
Item 6	9	1	0.9
Item 7	10	0	1.0
Item 8	9	1	0.9
Item 9	10	0	1.0
Item 10	10	0	1.0
Item 11	10	0	1.0

**Table 3:** Each item and overall content validity of the expert evaluation

4 Domain Sport PROM items	Number of selecting 3/4 level	Number of selecting 1/2 level	Experts in Agreement	I-CVI
Item 1	8	2	8	0.8
Item 2	10	0	10	1
Item 3	9	1	9	0.9
Item 4	9	1	9	0.9
Item 5	10	0	10	1
Item 6	10	0	10	1
Item 7	10	0	10	1
Item 8	10	0	10	1
Item 9	10	0	10	1
Item 10	10	0	10	1
Item 11	10	0	10	1

#### Step 5. Prefinal testing among the patients

A total of 20 patients with sports injuries who underwent surgical treatment and postoperative rehabilitation in the Sports Medicine Department of Huashan Hospital were selected to evaluate whether the translation was understandable (Table 4). All 20 patients were native Chinese speakers and fluent in written and written English. Patients used"yes"and"no"responses, and 80% of patients needed to select"yes"for each entry. Otherwise, the entry be adjusted according to the opinion of the patient. After this step, the final version of the 4 Domain Sport PROM Chinese version was obtained (Appendix 2).

**Table 4:** Bilingual evaluation of translation accuracy

4 Domain Sport PROM items	Number of “Yes”	Number of “No”	“Yes” Ration
Item 1	20	0	1.0
Item 2	19	1	0.95
Item 3	19	1	0.95
Item 4	19	1	0.95
Item 5	20	0	1.0
Item 6	20	0	1.0
Item 7	20	0	1.0
Item 8	20	0	1.0
Item 9	20	0	1.0
Item 10	20	0	1.0
Item 11	20	0	1.0

#### Step 6 Reliability and consistency testing

The research team recruited 120 patients who had undergone sports medicine surgery at the Department of Sports Medicine at Huashan Hospital affiliated to Fudan University. Enrolled subjects were required to take the test twice (one week between fill-in intervals). The 4 Domains Sports PROM reliability analysis was performed using Pearson correlation coefficient, ICC and Kappa values. ICC was calculated for the continuous variable Items, and ICC tests were performed separately for 4 different Domains. For categorical variable Items, Kappa values were used to calculate consistency. The Cronbach's α coefficient was used to reflect internal consistency (Table 5).

**Table 5:** Reliability and internal consistency of items of 4 Domains Sport PROM with presentation of mean values, standard deviation (SD), Pearson correlation, kappa and Cronbach’s alpha

4 Domain Sport PROM items	Mean(SD)		Pearson Correlation(*P* value)	Cronbach’s alpha excluded	Kappa (*P* value)
	Test	Retest			
Item 1	8.05(2.81)	8.38(2.44)	0.85(0.00)	0.69	-
Item 2	1.11(0.36)	1.11(0.36)	0.94(0.00)	-	0.90(0.00)
Item 3	7.81(2.65)	8.55(2.28)	0.83(0.00)	0.62	-
Item 4–1	0.86(0.35)	0.80(0.40)	0.69(0.00)	-	0.68(0.00)
Item 4–2	0.29(0.46)	0.31(0.46)	0.56(0.00)	-	0.56(0.00)
Item 4–3	0.73(0.45)	0.70(0.46)	0.57(0.00)	-	0.57(0.00)
Item 4–4	0.63(0.47)	0.66(0.48)	0.75(0.00)	-	0.75(0.00)
Item 4–5	0.60(0.50)	0.63(0.48)	0.51(0.00)	-	0.51(0.00)
Item 4–6	0.13(0.34)	0.18(0.38)	0.72(0.00)	-	0.71(0.00)
Item 4–7	0.15(0.36)	0.15(0.36)	0.67(0.00)	-	0.67(0.00)
Item 5	7.92(2.69)	8.54(2.29)	0.85(0.00)	0.61	-
Item 6	9.51(2.03)	9.47(1.72)	0.77(0.00)	0.70	-
Item 7	2.51(0.95)	2.29(1.01)	0.46(0.00)	-	0.48(0.00)
Item 8	9.77(1.70)	9.71(1.24)	0.48(0.00)	0.71	-
Item 9	9.18(1.72)	9.23(1.66)	0.77(0.00)	0.72	-
Item 10	10.37(1.04)	10.10(1.01)	0.65(0.00)	0.72	-
Item 11	9.63(1.65)	9.53(1.82)	0.66(0.00)	0.72	-
ICC(95%CI)	1 st Domain ICC (95%CI)	2nd Domain ICC (95%CI)	3rd Domain ICC (95%CI)	4 th Domain ICC (95%CI)	Cronbach’s alpha
0.72(0.64–0.79)	0.72(0.59–0.80)	0.61(0.43–0.73)	0.55(0.36–0.69)	0.63(0.46–0.74)	0.72

## Discussion

The main finding of this study was that the cross-cultural validation of the 4-Domain Sports PROM (4-DSP) into Chinese was adequately understandable in all questionnaire domains, and 97% of the experts considered that the translation accuracy was understandable; each item and overall content validity showed 96% agreement, the bilingual translation accuracy was rated as 98.5%, presenting a global Cronbach's alpha of 0.72.

The patients'voices and opinions on received treatment play an essential role in developing and refining a management plan of treatment approaches called Patient-Reported Outcomes Measures – PROM. [19–21] Therefore, it is undeniable that PROM have come to the forefront as a valuable tool to assess, investigate, and compare outcomes, boosting changes and improving treatment. [3, 12, 19, 22]

In orthopaedics, different outcome tools have been validated and used in clinical practice, most of them focusing on registering measurements of range of motion, joint stability, muscle strength and radiological assessment [7–10], factors that do not capture the patient's perception of the whole received treatment.

Furthermore, most have no target population and are applied equally to athletes or sedentary people, i.e. present individuals with different attitudes and perspectives toward life. [22, 23]

The apparent void of PROM tailored to assess athletes and regular sports practitioners [11] has become the starting point develop a PROM tailored for Sports Medicine – the 4-Domain Sports PROM.

The 4-DSP is an 11-item questionnaire designed to assess the athletic population, where three critical pieces of information about their level of sports practice, main physical demands in sports practice, and complaints after injury capture the athlete's baseline reported information and, at the same time serves to guide him/her to reply the two-graded questions from each one of the four domains. [15]

The 4-DSP is a multilingual project. [15–18] Its easy applicability in clinical practice has triggered our interest in expanding its use to the Chinese language given China's relevance to sports medicine and the fact that Chinese is one of the most widely spoken languages in the world.

The transcultural adaptation of 4-DSP into Chinese followed a strict process of content validation, which was obtained through the assessment of the instrument using a reliable and reproducible method as described in the literature. The Expert committee review identified excellent understandable accuracy in translation and each item and overall content validity.

The prefinal testing performed among 20 patients who were native Chinese speakers and fluent in English also showed a rate of 9.85 accuracy of bilingual translation assessment. The analysis of reliability and consistency enrolled 120 patients who had undergone sports medicine surgery. The test was applied twice (test and retest) and showed satisfactory reliability index levels in all domains, reflecting both the degree of correction and agreement between measurements.

The Chinese cross-cultural adaptation and validation from the original English version of the 4-DSP presented a global Cronbach´s alfa value of 0.63, an acceptable reliability value and internal consistency of this questionnaire version.

## Conclusion

The Chinese cross-cultural adaptation and validation of the original English version of the 4-DSP questionnaire proved reproducible and properly understandable in all four domains. It can safely and reliably assess treatment outcomes for sports injuries in Chinese-speaking patients and is a helpful tool to collect and assess athletic population data.

## Supplementary Information

Supplementary Material 1.

Supplementary Material 2.

## Data Availability

The datasets generated during and/or analysed during the current study are available throughout the manuscript.

## Abbreviations

4-DSP: 4-Domain Sport PROM
PROM: Patient-Reported Outcomes Measures
FAI: Femoroacetabular Impingement
ACL: Anterior Cruciate Ligament
PCL: Posterior Cruciate Ligament
MCL: Medial Collateral Ligament
IKDC: International Knee Documentation Committee
DASH: Disabilities of the Arm, Shoulder, and Head
ICC: Intraclass Correlation Coefficient
TA: Translatability Assessment
PRO: Patient-Reported Outcome
