# Supplementary material for: Translation, cross-cultural adaptation, and validation of the Chinese version of the 4 domain sports prom
Source: J Orthop Surg Res. 2025 May 19;20:487. doi: 10.1186/s13018-025-05882-1 (PMC12090455; doi:10.1186/s13018-025-05882-1)
Supplement: Supplementary file 1 — Supplementary Material 1. [file 13018_2025_5882_MOESM1_ESM.docx]

APPENDIX 1

PROM for Sports Medicine – Survey

**4-Domain Sports PROM – English (original version)**

1st Domain – uninjured baseline status (patient’s report)

**1. Does sport activity influence your quality of life?**

**^
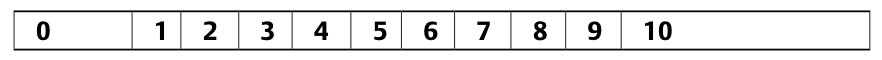
^None**  **high influence**

2) Considering your sports modality, **what is your level of competition**?

(1) recreational (2) regional (3) national (4) international

3) How **motivating** (positive activity) is **sports activity for you**?

**^
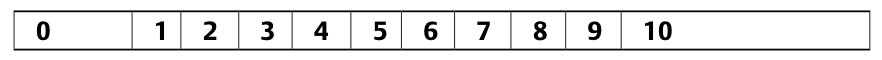
^None** **high influence**

4) What are **the main physical demands** in your sport activity?

(1) running (2) kicking (3) jumping (4) changing direction

(5) acceleration/deceleration (6) throwing (7) others

2nd DOMAIN - injury status (quality of life and sports performance)

5) How much did this **injury influence your quality of life**?

**^
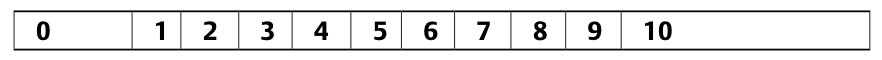
^None** **high influence**

6) Considering the **main physical demand reported**, how much did this injury influence your **sports performance**?

**^
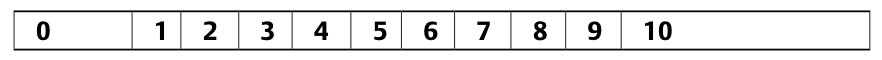
^None** **high influence**

7) What were your **most important physical complaints** (symptoms) after this injury?

(1) pain (2) joint stability (3) decrease of range of motion (4) loss of strength

**3rd DOMAIN - patient’s expectations**

8) After talking (discussing) with your doctor, did **you really understand** your injury?

**^
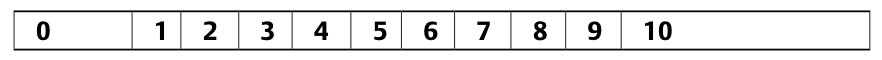
^no way** **no doubt**

9) Did you **expect to return to the same level of sports activity**?

**^
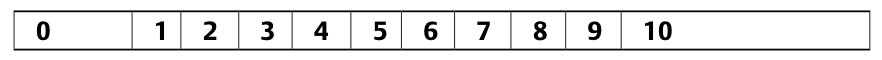
^no** **I’m very confident**

4th DOMAIN - Treatment and postoperative results

10) How did **you analyze your postoperative care**?

**^
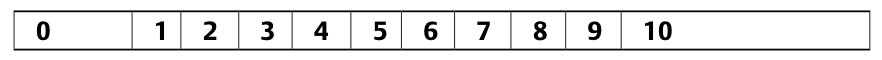
^very bad excellent**

11) Regarding your injury, how is **your feeling** (psychological status) **at the end of treatment** (final postoperative results)?

**^
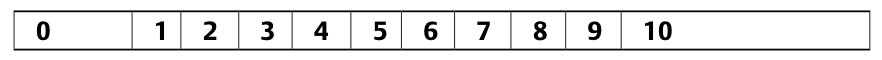
^very bad excellent**
