# Supplementary material for: Translation, cross-cultural adaptation, and validation of the Chinese version of the 4 domain sports prom
Source: J Orthop Surg Res. 2025 May 19;20:487. doi: 10.1186/s13018-025-05882-1 (PMC12090455; doi:10.1186/s13018-025-05882-1)
Supplement: Supplementary file 2 — Supplementary Material 2. [file 13018_2025_5882_MOESM2_ESM.docx]

APPENDIX 2

**4 Domain Sports PROM Chinese Version（Final Version）**

4模块运动相关患者报告结局量表

模块1-未受伤的基线水平（患者报告）

1. 体育运动会影响您的生活质量吗？

| 0 | 1 | 2 | 3 | 4 | 5 | 6 | 7 | 8 | 9 | 10 |
| --- | --- | --- | --- | --- | --- | --- | --- | --- | --- | --- |

无影响 很大影响

1. 参考您的运动能力，您的竞技水平为？

（1）休闲娱乐水平 （2）省市级 （3）国家级 （4）国际级

1. 您参与体育运动的动机（主动运动）有多强烈？

| 0 | 1 | 2 | 3 | 4 | 5 | 6 | 7 | 8 | 9 | 10 |
| --- | --- | --- | --- | --- | --- | --- | --- | --- | --- | --- |

无 非常强烈

1. 在您从事的体育运动中，哪一些是主要的运动能力需求？（此题为多选题）
2. 跑步 （2）踢 （3）跳跃 （4）变向 （5）加速/减速 （6）投掷 （7）其他

模块2-损伤状况（生活质量和运动表现）

1. 该损伤对您的生活质量有多大影响？

| 0 | 1 | 2 | 3 | 4 | 5 | 6 | 7 | 8 | 9 | 10 |
| --- | --- | --- | --- | --- | --- | --- | --- | --- | --- | --- |

无 很大影响

1. 参考已填写的主要运动能力需求，该损伤对您的运动表现有多大影响？

| 0 | 1 | 2 | 3 | 4 | 5 | 6 | 7 | 8 | 9 | 10 |
| --- | --- | --- | --- | --- | --- | --- | --- | --- | --- | --- |

无 很大影响

1. 这次损伤之后您最主要的不适（症状）是什么？

（1）疼痛 （2）关节不稳 （3）活动度下降 （4）力量下降

模块3-患者的期待

1. 在与您的医生交流（讨论）后，您切实了解您的伤病情况吗？

| 0 | 1 | 2 | 3 | 4 | 5 | 6 | 7 | 8 | 9 | 10 |
| --- | --- | --- | --- | --- | --- | --- | --- | --- | --- | --- |

不明白 无疑问

1. 您期待重返伤前的运动水平吗？

| 0 | 1 | 2 | 3 | 4 | 5 | 6 | 7 | 8 | 9 | 10 |
| --- | --- | --- | --- | --- | --- | --- | --- | --- | --- | --- |

不期待 非常有信心

模块4-治疗和术后结局

1. 对于您的术后医疗照护（包括医生、康复师、护士、教练员等多角色的团队合作），您作何评价？

| 0 | 1 | 2 | 3 | 4 | 5 | 6 | 7 | 8 | 9 | 10 |
| --- | --- | --- | --- | --- | --- | --- | --- | --- | --- | --- |

非常差 非常棒

1. 基于您的损伤，在治疗结束时（最终治疗结果）您的感觉（心理状态）如何？

| 0 | 1 | 2 | 3 | 4 | 5 | 6 | 7 | 8 | 9 | 10 |
| --- | --- | --- | --- | --- | --- | --- | --- | --- | --- | --- |

非常差
